# What might explain deprivation-specific differences in the excess hazard of breast cancer death amongst screen-detected women? Analysis of patients diagnosed in the West Midlands region of England from 1989 to 2011

**DOI:** 10.18632/oncotarget.10255

**Published:** 2016-06-23

**Authors:** Melanie Morris, Laura M. Woods, Bernard Rachet

**Affiliations:** ^1^ Cancer Research UK Cancer Survival Group, Faculty of Epidemiology and Population Health, Department of Non-Communicable Disease Epidemiology, London School of Hygiene & Tropical Medicine, London WC1E 7HT, UK

**Keywords:** breast cancer, socioeconomic inequalities, survival analysis, early diagnosis, population-based

## Abstract

**Background:**

Breast cancer survival is higher in less deprived women, even amongst women whose tumor was screen-detected, but reasons behind this have not been comprehensively investigated.

**Methods:**

The excess hazard of breast cancer death in 20,265 women diagnosed with breast cancer, followed up to 2012, was estimated for screen-detected and non-screen-detected women, comparing more deprived to less deprived women using flexible parametric models. Models were adjusted for individual and tumor factors, treatment received and comorbidity. For screen-detected women, estimates were also corrected for lead-time and overdiagnosis.

**Results:**

The excess hazard ratio (EHR) of breast cancer death in the most deprived group, adjusted only for age and year of diagnosis, was twice that of the least deprived among screen-detected women (EHR=2.12, 95%CI 1.48-2.76) and 64% higher among non-screen-detected women (EHR=1.64, 95%CI 1.41-1.87). Adjustment for stage at diagnosis lowered these estimates by 25%. Further adjustment had little extra impact. In the final models, the excess hazard for the most deprived women was 54% higher (EHR=1.54, 95%CI 1.10-1.98) among screen-detected women and 39% higher (EHR=1.39, 95%CI 1.20-1.59) among non-screen-detected women.

**Conclusion:**

A persistent socio-economic gradient in breast cancer-related death exists in this cohort, even for screen-detected women. The impact of differential lifestyles, management and treatment warrant further investigation.

## INTRODUCTION

In the UK, deprived women have lower breast cancer survival [1–5]. This has also been found to be the case in other countries [6–9]. The overall difference in breast cancer survival between affluent and deprived women was not modified in the UK by introduction of screening in 1989 [10] and remains despite a mature screening program and a recent narrowing of the survival gap [11]. The discrepancy in survival by deprivation remains a key focus of public health policy [12].

We have previously examined this cohort 20, 265 women diagnosed in the West Midlands region of England from 1989 to 2011 [5]. We reported higher breast cancer survival in less deprived women amongst both women whose cancer was detected following symptoms, as well as amongst women whose tumor was screen-detected, and thus likely to be asymptomatic. In the same analysis, we showed no differences in breast cancer survival between ethnic groups. Together, these findings suggest that, although screening confers a survival benefit to all women, there are still wide, unexplained disparities in breast cancer survival by deprivation.

Whereas socio-economic inequalities overall may partly be explained by later presentation, leading to more advanced disease [13–15] it is less probable that stage of disease explains socio-economic differences amongst screen-detected women who are asymptomatic at presentation. Reasons for this residual socio-economic gradient in breast cancer survival in screen-detected women have not yet been comprehensively investigated. Screen-detected women provide an ideal opportunity to examine these since the differences in stage at presentation between more and less deprived women is substantially reduced.

Here we model the excess hazard of breast cancer death (i.e. the hazard of death on top of the expected hazard of death, therefore related to breast cancer) amongst more deprived compared to more affluent women, in order to examine whether variations in tumor characteristics, pre-existing comorbidities or surgery might explain deprivation-specific inequalities in breast cancer survival where women have been diagnosed asymptomatically.

## RESULTS

### Cohort characteristics

We included in the analysis 20,265 women who had a first primary malignant breast tumor (mean age 57.5 years, standard deviation=5.0, 96.4% of those eligible) and who were continuously eligible for screening from the age of 50 onwards. We compared women whose breast cancer was screen-detected (n=10,502, 51.8%) to women who were not screen-detected (n=9,763, 48.2%).

Most women were diagnosed with localized disease (60.0%) and were alive at the end of follow-up (81.6%, Table 1). There were more women in the less deprived categories (e.g. 22.1% in the least deprived compared with 17.0% in the most deprived). Women in the more deprived categories were less likely to have local disease (58.0% in the most deprived compared to 62.2% in the least deprived women, χ^2^ p-value<0.001) or first surgery within 2 weeks of diagnosis (31.3% compared to 37.2%, p<0.001) and more likely to have larger tumors (6.2% compared to 4.7%, p<0.001), comorbidities (7.3% compared to 4.0%, p<0.001) and be deceased by the end of follow-up (21.8% compared to 16.2%, p<0.001). They were also slightly older (e.g. 11.6% compared to 9.3% in the oldest age group, p=0.04). Grade did not vary across deprivation categories (p=0.260), nor did screening history (p=0.120).

**Table 1:** Cohort and tumor characteristics by deprivation category, n (%) and mean [standard deviation] for continuous variables

N=20,265	Least deprived 1	2	3	4	Most deprived 5	Total	p-value (χ^2^)
**Age at diagnosis^a^**							
** Mean [sd]**	**57.2 [4.9]**	**57.6 [5.1]**	**57.5 [5.0]**	**57.5 [5.1]**	**57.7 [5.1]**	**57.5 [5.0]**	0.040
** 50-54**	1,576 (35.2)	1,556 (33.7)	1,407 (33.7)	1,218 (34.3)	1,122 (32.5)	**6,879 (33.9)**	
** 55-59**	1,442 (32.2)	1,412 (30.6)	1,307 (31.3)	1,084 (30.6)	1,089 (31.6)	**6,334 (31.3)**	
** 60-64**	1,042 (23.3)	1,141 (24.7)	990 (23.7)	847 (23.9)	840 (24.3)	**4,860 (24.0)**	
** 65-70**	418 (9.3)	505 (10.9)	472 (11.3)	397 (11.2)	400 (11.6)	**2,192 (10.8)**	
**Vital status at end of follow up**							
**Alive**	3752 (83.8)	3854 (83.5)	3421 (81.9)	2810 (79.2)	2699 (78.2)	**16,536 (81.6)**	<0.001
**Dead**	726 (16.2)	760 (16.5)	755 (18.1)	736 (20.8)	752 (21.8)	**3,729 (18.4)**	
**Screening history^b^**							
**SD**	2,320 (51.8)	2,380 (51.6)	2,232 (53.4)	1,830 (51.6)	1,740 (50.4)	**10,502 (51.8)**	0.120
**Not SD**	2,158 (48.2)	2,234 (48.4)	1,944 (46.6)	1,716 (48.4)	1,711 (49.6)	**9,763 (48.2)**	
**Period of diagnosis^a^**							
**1989-1994**	200 (4.5)	167 (3.6)	161 (3.9)	136 (3.8)	137 (4.0)	**801 (4.0)**	<0.001
**1995-2000**	934 (20.9)	866 (18.8)	787 (18.8)	651 (18.4)	600 (17.4)	**3,838 (18.9)**	
**2001-2006**	1,765 (39.4)	1,781 (38.6)	1,555 (37.2)	1,383 (39.0)	1,300 (37.7)	**7,784 (38.4)**	
**2007-2011**	1,579 (35.3)	1,800 (39.0)	1,673 (40.1)	1,376 (38.8)	1,414 (41.0)	**7,842 (38.7)**	
**Extent of disease**							
**Local**	2,786 (62.2)	2,795 (60.6)	2,539 (60.8)	2,048 (57.8)	2,002 (58.0)	**12,170 (60.1)**	<0.001
**Regional**	1,305 (29.1)	1,435 (31.1)	1,303 (31.2)	1,169 (33.0)	1,146 (33.2)	**6,358 (31.4)**	
**Distant**	98 (2.2)	97 (2.1)	78 (1.9)	106 (3.0)	104 (3.0)	**483 (2.4)**	
***Missing***	*289 (6.5)*	*287 (6.2)*	*256 (6.1)*	*223 (6.3)*	*199 (5.8)*	***1,254 (6.2)***	
**Histological group**							
**Ductal**	3,274 (73.1)	3,297 (71.5)	2,962 (70.9)	2,475 (69.8)	2,497 (72.4)	**14,505 (71.6)**	0.001
**Lobular**	673 (15.0)	784 (17.0)	690 (16.5)	581 (16.4)	486 (14.1)	**3,214 (15.9)**	
**All other**	509 (11.4)	512 (11.1)	503 (12.0)	463 (13.1)	442 (12.8)	**2,429 (12.0)**	
***Missing***	*22 (0.5)*	*21 (0.5)*	*21 (0.5)*	*27 (0.8)*	*26 (0.8)*	***117 (0.6)***	
**Grade of tumor**							
**I**	932 (20.8)	932 (20.2)	858 (20.5)	692 (19.5)	671 (19.4)	**4,085 (20.2)**	0.260
**II**	1,897 (42.4)	2,027 (43.9)	1,778 (42.6)	1,537 (43.3)	1,436 (41.6)	**8,675 (42.8)**	
**III**	1,292 (28.9)	1,286 (27.9)	1,222 (29.3)	1,019 (28.7)	1,072 (31.1)	**5,891 (29.1)**	
***Missing***	*357 (8.0)*	*369 (8.0)*	*318 (7.6)*	*298 (8.4)*	*272 (7.9)*	***1,614 (8.0)***	
**Size of tumor (mm) ^a, c^**							
**Mean [sd]**	**20.1 [15.4]**	**20.5 [15.7]**	**21.1 [17.5]**	**21.4 [17.1]**	**22.4 [17.4]**	**21.0 [16.6]**	<0.001
**0 - 20**	2,490 (55.6)	2,486 (53.9)	2,242 (53.7)	1,839 (51.9)	1,707 (49.5)	**10,764 (53.1)**	
**20 - 50**	1,380 (30.8)	1,484 (32.2)	1,387 (33.2)	1,183 (33.4)	1,251 (36.3)	**6,685 (33.0)**	
**50 mm+**	211 (4.7)	237 (5.1)	226 (5.4)	199 (5.6)	215 (6.2)	**1,088 (5.4)**	
***Missing***	*397 (8.9)*	*407 (8.8)*	*321 (7.7)*	*325 (9.2)*	*278 (8.1)*	***1,728 (8.5)***	
**Charlson score**							
**0**	4,301 (96.0)	4,444 (96.3)	3,991 (95.6)	3,345 (94.3)	3,199 (92.7)	**19,280 (95.1)**	<0.001
**1**	77 (1.7)	90 (2.0)	99 (2.4)	100 (2.8)	151 (4.4)	**517 (2.6)**	
**2**	92 (2.1)	71 (1.5)	68 (1.6)	84 (2.4)	77 (2.2)	**392 (1.9)**	
**3**	8 (0.2)	9 (0.2)	18 (0.4)	17 (0.5)	24 (0.7)	**76 (0.4)**	
**Surgery**							
**No**	288 (6.4)	306 (6.6)	252 (6.0)	289 (8.2)	279 (8.1)	**1,414 (7.0)**	<0.001
**Yes**	4,190 (93.6)	4,308 (93.4)	3,924 (94.0)	3,257 (91.8)	3,172 (91.9)	**18,851 (93.0)**	
**Total**	**4,478 (100)**	**4,614 (100)**	**4,176 (100)**	**3,546 (100)**	**3,451 (100)**	**20,265 (100)**	
**Time from diagnosis to first surgery^a^ (among those who had surgery, n=18,851)**							
**Mean [sd] (days)**	**21.6 [23.6]**	**22.6 [24.9]**	**22.1 [23.4]**	**23.5 [25.5]**	**23.5 [25.2]**	**22.6 [24.5]**	
**0 – 2 weeks**	1,557 (37.2)	1,530 (35.5)	1,313 (33.5)	1,036 (31.8)	992 (31.3)	**6,428 (34.1)**	<0.001
**2 - 6 weeks**	2,317 (55.3)	2,408 (55.9)	2,336 (59.5)	1,934 (59.4)	1,914 (60.3)	**10,909 (57.9)**	
**6 + weeks**	289 (6.9)	341 (7.9)	250 (6.4)	270 (8.3)	248 (7.8)	**1,398 (7.4)**	
***Missing***	27 (0.6)	29 (0.7)	25 (0.6)	17 (0.5)	18 (0.6)	***116 (0.6)***	
**Total**	**4,190 (100)**	**4,308 (100)**	**3,924 (100)**	**3,257 (100)**	**3,172 (100)**	**18,851 (100)**	

a Fitted as a continuous variable in the model

b SD=screen detected, not SD=not screen-detected

c Grouped here according to TNM criteria: T1 >=20 mm, T2 20mm-50 mm, T3 >=50 mm [45].

### Excess hazard of breast cancer death

Among non-screen-detected women, the excess hazard of death from breast cancer, adjusted only for age and year of diagnosis, within five years of diagnosis was 64% higher in the most deprived group compared with the least deprived group (EHR=1.64, 95%CI 1.41-1.87) (Table 2) and more than double among screen-detected women (EHR=2.12, 95%CI 1.48-2.76). These hazard ratios reduced by around 25% after adjustment for stage of disease. Tumor characteristics and treatment did not have any notable impact. The inclusion of comorbidity reduced the excess hazard ratios slightly in both groups.

**Table 2:** Screening-specific excess hazard ratios at five years after diagnosis for each model in turn, comparing the most deprived to the least deprived group (reference)

Model		Variable	Form of variable in the model		Excess Hazard Ratios (95% CIs)	
			Non-screen-detected	Screen-detected	Non-screen-detected (n=8,962)	Screen-detected (n=8,541.5)^a^
**1**	= Baseline (3 df for baseline hazard)	Age (continuous)	Non-linear (2 df); time- varying effect (1df)	Linear	1.64 (1.41-1.87)	2.12 (1.48-2.76)
		Year of diagnosis (continuous)	Linear	Linear		
		Deprivation (categorical)	Linear	Linear		
**2**	= 1 + stage of disease at diagnosis	Extent of disease (categorical)	Linear	Linear	1.47 (1.26-1.68)	1.66 (1.18-2.13)
		Tumor size (continuous)	Linear	Linear		
**3**	= 2 + tumor characteristics	Histology (categorical)	Linear	Linear	1.45 (1.24-1.65)	1.65 (1.18-2.12)
**4**	= 3 + treatment	Surgery (categorical)	Linear	Linear	1.44 (1.25-1.64)	1.66 (1.19-2.13)
		Time to surgery (continuous)	Linear	*Not included*		
**Final model**	= 4 + Comorbidity	Charlson score (continuous)	Linear	Linear	1.39 (1.20-1.59)	1.54 (1.10-1.98)

a The number of women in the screen-detected group is the mean of the 10 imputed datasets and excludes women considered to be overdiagnosed.

There was a time-varying effect of age in the non-screen-detected group. Time to surgery did not improve the fit of the final model for the screen-detected group but was included for non-screen-detected women. No interactions were found. In the final models, the excess hazard for women in the most deprived group was 39% higher (EHR=1.39, 95%CI 1.20-1.59) among non-screen-detected women, and 54% higher (EHR=1.54, 95%CI 1.10-1.98) for the screen-detected.

### Sensitivity analysis

The sensitivity analysis suggested that use of the complete case data underestimated the excess hazard which was adjusted only for age and year of diagnosis, (by 5% in non-screen-detected and 8% in screen-detected). The analysis also showed that results for the final model were likely to be robust; when all the less deprived women had favorable tumors and all the more deprived had unfavorable, excess mortality remained higher for deprived women, by 19% in non-screen-detected and 49% in screen-detected women (Table 3).

**Table 3:** Sensitivity analysis of screening-specific excess hazard ratios at five years after diagnosis, comparing the most deprived to the least deprived group (reference)

	Non-screen-detected		Screen-detected	
	Baseline model	Final model	Baseline model	Final model
**Complete case**	**n= 8,962**		**n= 8,541.5^a^**	
	1.64 (1.41-1.87)	1.39 (1.20-1.59)	2.12 (1.48-2.76)	1.54 (1.10-1.98)
**Sensitivity analysis^b^**	**n= 9,624**		**n= 9012.9^a^**	
	1.69 (1.46-1.92)	1.19 (1.03-1.35)	2.20 (1.55-2.84)	1.49 (1.06-1.91)

a The number of women in the screen-detected group is the mean of the 10 imputed datasets and excludes women considered to be overdiagnosed.

b Less deprived women with missing data assumed to have small, localized tumors whilst more deprived women with missing data are assumed to have large tumors with distant spread.

## DISCUSSION

We have demonstrated a socio-economic gradient in breast cancer-related death in the West Midlands among women eligible for screening regardless of whether the woman's breast cancer was screen-detected or not. This difference persists after adjustment for stage of disease at diagnosis (including size of tumor), histology, surgery (including time to surgery) and pre-existing comorbidities.

The data used for this study come from a center of excellence, individually linked for each woman to various data sources with good completeness on most variables. Uniquely and importantly, our cohort for analysis included only women aged 50 to 70 years old who had been continuously eligible for screening, in order to exclude women diagnosed within the screening age range but who had no opportunity for the cancer to be screen-detected at the time of diagnosis.

We used robust, up-to-date statistical techniques to estimate the differences in the excess hazard of cancer death between more affluent and more deprived women. We used deprivation- and ethnicity-adjusted life tables in order to obtain the most accurate correction for background mortality [16, 17]. We also corrected for lead time bias [18, 19] and overdiagnosis, which allowed a direct comparison between screen-detected and non-screen-detected women [20, 21]. Studies in other countries have looked at the impact of screening and socio-economic status on breast cancer survival, and come to similar conclusions to this study [7, 8, 22]. However, none have used the net survival framework (either net survival or the excess hazard of breast cancer death), nor adjusted for lead-time or overdiagnosis as we do here. As such, they may have overestimated the difference between deprivation groups. In addition, none of these previous studies has taken place in England and thus they have not examined the particular context of the NHS Breast Screening Programme.

Information on individual deprivation status is unobtainable from any of the data sources available. We thus defined deprivation ecologically using the validated score temporally closest to the women's date of diagnosis, in order to measure as closely as possible the impact of deprivation on diagnosis and cancer management. These ecological data provide only a proxy measure of the underlying variable of interest. They are susceptible to the ecological fallacy, as well as to temporal change. In reality it is probable that these ecologically-based analyses underestimate the true underlying differences between women of different socio-economic status. This could be examined in a dataset where both ecological and individual measures of deprivation could be derived.

We examined the potential impact of missing data upon our results via a sensitivity analysis. This suggests that the differences we observe in the excess hazard of breast cancer death are robust to the (unknown) distributions of missing data. In reality, we consider it probable that the true (unmeasurable) excess hazard ratio falls between the complete-case and sensitivity estimate.

We determined cancer stage using both extent of disease and tumor size at diagnosis. Together these variables resulted in a sizeable reduction in excess mortality estimates for both screened and non-screen-detected women. Although extent of disease was strongly associated with deprivation in both screening groups in this study, the proportion of regional and advanced tumors was particularly high in the most deprived category of screen-detected women. These women also had tumors that were larger. This raises the possibility that the more deprived women with symptoms may use screening as an entry point more frequently than the less deprived women with symptoms; that is, attend ‘routine’ screening when they suspect cancer or are experiencing breast symptoms, in place of visiting their GP. Qualitative studies could explore this hypothesis further by examining women's motivations for attending screening by deprivation. Detailed examination of screening attendance patterns amongst all women could also be conducted, for example, examining the proportion of appointments attended on time, late, or not at all amongst more affluent compared to more deprived groups. Additionally, symptom awareness may be lower among the more deprived women [23, 24], leading to more women with regional or distant disease being diagnosed via the screening service. Either way, our data suggest that stage of disease, and thus timeliness of presentation, play a role in deprivation-specific differences in the excess hazard, even among screen-detected women, but that important deprivation differences still remain.

We were unable to adjust for other tumor factors including hormone receptor status as these variables were too incomplete. Surprisingly, surgical treatment received did not further explain differences in the excess hazard in either group, where previously this has been shown to be influential [8]. Additionally, time to surgery was not found here to improve the fit of any of the models for the screen-detected group, similar to the findings of a recent smaller study [25]. However, the measure available was not very discriminatory, partly because almost all women had surgery, but also because information on hormone therapy, chemotherapy and radiotherapy were too incomplete to use. Administration of neo-adjuvant hormone therapy, not detectable in our data, might also have extended time to surgery for some women.

Comorbidities reduced the deprivation gap in the excess hazard by only a small amount, consistent with the fact that the cohort were ostensibly healthy younger women up to age 70. The impact of comorbidities upon socio-economic differences may be greater among older women [6, 8].

Our results suggest that factors other than stage of disease, tumor characteristics, surgical treatment and comorbidity lead to socio-economic differences in the excess hazard of breast cancer death, and that early, asymptomatic diagnosis via screening does not eliminate them. Differential use or receipt of treatments in breast cancer must be considered as an important potential explanation for this, and have previously been shown to be associated with socio-economic differences in survival [14, 15, 26–28]. It has been comprehensively shown that where treatment is consistent (within clinical teams or within clinical trials) socio-economic differences are not apparent [29, 30]. Differences in treatment actually received might originate from differences in ease of travel to appointments, flexibility of work, other commitments or levels of social support [31–33]. Differences in nutrition, smoking, alcohol consumption or other health-related behaviors are also possible explanations, although evidence for the impact of these upon cancer survival differentials is mixed [14]. The possibility of more frequent use of the screening service by more deprived women as a point of entry also deserves further examination.

Deprivation status is the socio-demographic characteristic that most strongly impacts the survival of women in the UK with breast cancer, after age at diagnosis. In this study, we have shown that the more deprived women with breast cancer have a higher excess hazard of cancer death, irrespective of whether their cancer was screen-detected or not. Our data suggest that this pattern is not simply a product of differences in timely diagnosis as it occurs even among women diagnosed asymptomatically. Having accounted here for some patient and tumor factors, we have shown that early diagnosis through screening is not enough to eliminate inequalities, and that the influence of differential cancer management, treatment, as well as lifestyles or life challenges of breast cancer patients from more deprived communities warrants further investigation.

## MATERIALS AND METHODS

### Data

As in our previous study,[5] the cohort consisted of women diagnosed with a primary, invasive breast cancer during the period 1 April 1989–31 March 2011, aged 50–70 years at their diagnosis, who were continuously eligible for screening from the age of 50 up to either 65 or 70 years (if the screening service expanded) and who would have received their first invitation letter from their 50th birthday onwards. A total of 20,283 women were included after excluding 761 women (3.6% of those eligible). These exclusions mainly comprised women whose tumors were recurrences of earlier malignancies. The mean date of diagnosis of these women was 14^th^ August 2004. Cancer-related information on these individuals was obtained from the West Midlands Breast Screening Quality Assurance Reference Centre (Cancer Registry) [34, 35]. These data were individually linked to Hospital Episode Statistics and the National Breast Screening Service records. The vital status of all women was known at the close of follow-up on 31 July 2012, after which each woman's follow-up time was censored. The median follow-up time for all women was 5.77 years. For the 3,732 women who died during the study period median follow-up was 3.24 years.

Women whose breast cancer was not screen-detected included those whose last screening attendance had resulted in a negative screen and had not yet been invited to a subsequent screening (*interval cancer*; n=6,311, 31.1%); women whose cancer was diagnosed after having previously had a negative screen, but who had not attended their most recent screening appointment (*lapsed attenders*, n=1,142, 5.6%); and women who had never presented for screening (*non-attenders*, n=2,310, 11.4%).

Deprivation was measured using the income domain of the English indices of multiple deprivation (IMD) for 2004, 2007 or 2010 [36–38]. These ecologically-based scores are derived from routine administrative data pertaining to the years 2001, 2005 and 2008 respectively, and are calculated for each of the 32,482 Lower Super Output Areas as defined at the 2001 census (LSOAs, approximately 1,500 people). The scores are categorized according to the quintiles of their national distribution. Each woman was assigned to one of five deprivation levels on the basis of her address of residence and date of diagnosis. Those with missing information on deprivation were excluded from all analyses (18 women, 0.1%), resulting in 20,265 women in the analyses.

Information on tumor size, nodal involvement and presence of metastases was used to establish each woman's extent of disease at diagnosis, either localized (confined to the organ of origin), regional (spread to adjacent muscle, organ, fat, connective tissue or regional lymph nodes) or distant (distant metastases). Tumors were further categorized by their histology (ductal, lobular or all others) and grade (I, II or III).

Treatment was evaluated by surgery on the tumor within 6 months of diagnosis (yes/no), and time from diagnosis to first surgery. Information on radiology, chemotherapy and hormone therapy was too incomplete to be used (22.9%, 62.5% and 50.6% missing respectively).

### Adjustment for lead time bias and over-diagnosis

Lead time bias arises from the inflation of an individual woman's recorded survival due to the cancer being detected at an earlier point in time at screening, but with no associated improvement in prognosis. To account for this we applied the method established by Duffy *et al* [20]. Our approach has been described previously [5]. Briefly, for each patient, additional survival time due to screening, E(s), was estimated assuming an exponential distribution of survival times, and a mean sojourn time (from carcinogenesis to symptomatic detection) of 4 years. In order to account for the uncertainty associated with this value, we generated 10 separate data sets for the screen-detected group containing E(s)_1_, E(s)_2_ … E(s)_10_ assuming these values were exponentially distributed with a mean of E(s).

Tumors are considered over-diagnosed if they would not have been detected symptomatically during the study period or during the woman's predicted lifetime [39]. To account for overdiagnosis we excluded tumor records where the value of E(s) exceeded the woman's actual observed survival time, was after 31st March 2011, or in excess of her life expectation at diagnosis (as defined in the life tables).

### Modelling of excess hazard ratio

We fitted flexible parametric log-cumulative excess hazard regression models [40] to estimate the excess hazard ratio which compares the excess hazard of breast cancer death (the analog of net survival) in less deprived women to that in more deprived women during the first five years after diagnosis. The excess hazard is the hazard of death experienced by the cancer patients on top of their expected hazard of death. We used ethnic-specific life tables for England and Wales adjusted for deprivation [41] to estimate expected mortality. Initial models were fitted separately for non-screen detected women, and for the 10 separate datasets derived for screen-detected women. These models included, a priori, age, year of diagnosis and deprivation category. We tested the linearity of age, year of diagnosis and deprivation category using Akaike's Information Criterion (AIC). A reduction of 3 or more in the AIC between successive models was taken to show a better model fit [42]. We evaluated the time-varying effect of each variable by the inclusion of restricted cubic splines. We subsequently adjusted for the stage of the tumor (using the variables extent of disease and tumor size, including the time-varying effect of extent), tumor characteristics (using histological group), treatment (using surgery and time from diagnosis to first surgery) and the presence of comorbidity (using the Charlson score [43]). Size of tumor and time from diagnosis to first surgery were transformed prior to inclusion in the models. Grade of tumor was excluded since there was no evident association with deprivation (Table 1). We evaluated interactions between age and deprivation, extent of disease and deprivation, and period of diagnosis and deprivation.

This process resulted in one model final specification for the non-screen-detected group and 10 different specifications for the screen-detected group. Eight out of these 10 were identical and thus defined the final specification (Appendix A).

We derived final estimates of the excess hazard ratio in the most deprived compared to the least deprived women using the final specifications. For the screen-detected group, the final model specification was fitted to each of the 10 sets of data. We then applied the rules established by Rubin [44] for the re-combination of estimates in a multiple-imputation setting to derive an overall estimate of the hazard ratio and its variance in the screen-detected group, adjusted for lead time bias and over-diagnosis (Appendix B).

### Sensitivity analyses for missing data

The initial models were derived using cases with complete records for all variables (93.1% among the non-screen-detected, 94.8% among the screen detected). We conducted a sensitivity analysis to examine the possible impact of these missing data on our estimates. Where a variable of interest had more than 1% missing data (extent of disease, tumor size; Table 1) we imputed values which assumed that the less deprived women with missing data had tumors with much better prognosis. Specifically, values for extent of disease for the less deprived women (categories 1 and 2) were imputed as localized and tumor size to the mean size of localized tumors (16.6mm), whilst among the more deprived women (categories 3, 4 and 5) extent of disease was imputed as distant and tumor size to the mean size of distant tumors (47.8mm). Variables with less than 1% of missing data (histology and time to first surgery) were not considered and women with missing information for these variables were excluded from the analyses (0.6% and 0.4% respectively).

## SUPPLEMENTARY MATERIALS APPENDIX


