# Supplementary material for: What might explain deprivation-specific differences in the excess hazard of breast cancer death amongst screen-detected women? Analysis of patients diagnosed in the West Midlands region of England from 1989 to 2011
Source: Oncotarget. 2016 Jun 23;7(31):49939–47. doi: 10.18632/oncotarget.10255 (PMC5226559; doi:10.18632/oncotarget.10255)
Supplement: Supplementary file 1 [file oncotarget-07-49939-s001.pdf]

What might explain deprivation-specific differences in the excess hazard of breast cancer death amongst screen-detected women? Analysis of patients diagnosed in the West Midlands region of England from 1989 to 2011

APPENDIX

	Set number	Age	Year	Deprivation	Extent of disease	Size of tumour	Histology	Surgery	Time to surgery	Charlson score	Non-linear	Time-varying
		Variables included									Effects included	
**Screen-detected**												
	1	Y	Y	Y	Y	Y	Y	Y	N	Y	None	Age (1 df)
	2*	Y	Y	Y	Y	Y	Y	Y	N	Y	None	None
	3*	Y	Y	Y	Y	Y	Y	Y	N	Y	None	None
	4	Y	Y	Y	Y	Y	Y	Y	N	Y	None	Deprivation (1 df)
	5*	Y	Y	Y	Y	Y	Y	Y	N	Y	None	None
	6*	Y	Y	Y	Y	Y	Y	Y	N	Y	None	None
	7*	Y	Y	Y	Y	Y	Y	Y	N	Y	None	None
	8*	Y	Y	Y	Y	Y	Y	Y	N	Y	None	None
	9*	Y	Y	Y	Y	Y	Y	Y	N	Y	None	None
	10*	Y	Y	Y	Y	Y	Y	Y	N	Y	None	None
**Non-screen-detected**												
		Y	Y	Y	Y	Y	Y	Y	Y	Y	Age (2 df)	Age (1 df)

Key: Y Variable included in the specification
NVariable not included in the specification
* Selected specification for screen-detected group (identical in 8 out of 10 datasets)

Appendix A: Model specifications derived from decision tree for screen-detected and non-screen detected women.

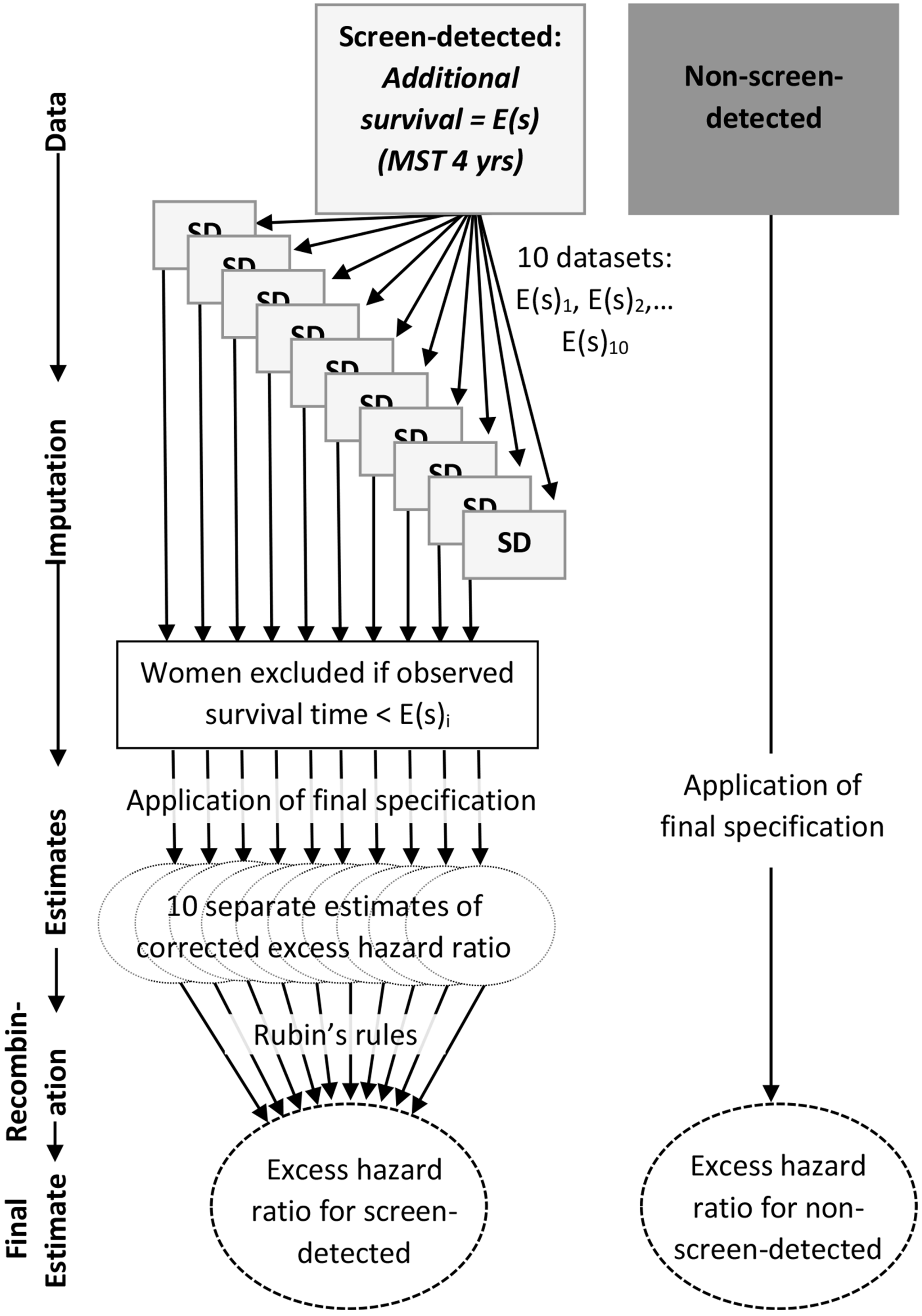

Appendix B: Derivation of final estimates for screen-detected and non-screen detected women.
